# Umbilical Cord Milking in Infants Born at <37 Weeks of Gestation: A Systematic Review and Meta-Analysis

**DOI:** 10.3390/jcm9041071

**Published:** 2020-04-09

**Authors:** Inmaculada Ortiz-Esquinas, Juan Gómez-Salgado, Julián Rodriguez-Almagro, Ángel Arias-Arias, Ana Ballesta-Castillejos, Antonio Hernández-Martínez

**Affiliations:** 1 Department of Obstetrics & Gynaecology, Alcázar de San Juan, 13600 Ciudad Real, Spain; inmaores@hotmail.com (I.O.-E.); antomatron@gmail.com (A.H.-M.); 2 Department of Sociology, Social Work and Public Health, University of Huelva, 21071 Huelva, Spain; jgsalgad@gmail.com; 3 Safety and Health Postgraduate Programme, Espíritu Santo University, Guayaquil 091650, Ecuador; 4 Department of Nursing, Faculty of Nursing of Ciudad Real, University of Castilla-La Mancha, 13071 Ciudad Real, Spain; 5 Research Support Unit, “Mancha-Centro” Hospital, Alcázar de San Juan, 13600 Ciudad Real, Spain; 6 Department of Obstetrics & Gynaecology, Hospital Talavera de la Reina, 45600 Toledo, Spain;ana.ballesta81@gmail.com

**Keywords:** Umbilical cord milking, delayed umbilical cord clamping, immediate umbilical cord clamping, preterm infants, systematic review and meta-analysis

## Abstract

Umbilical cord milking (UCM) could be an alternative in cases where delayed umbilical cord clamping cannot be performed, therefore our objective was to evaluate the effects of UCM in newborns <37 weeks’ gestation. In this systematic review and meta-analysis, we searched MEDLINE, EMBASE, CINAHL, the Cochrane Database of Clinical Trials, the clinicaltrails.gov database for randomized UCM clinical trials with no language restrictions, which we then compared with other strategies. The sample included 2083 preterm infants. The results of our meta-analysis suggest that UCM in premature infants can reduce the risk of transfusion (relative risk (RR)= 0.78 [95% confidence interval (CI),0.67–0.90]) and increase hemoglobin(pooled weighted mean difference (PWMD)= 0.89 g/L[95%CI 0.55–1.22]) and mean blood pressure (PWMD=1.92 mmHg [95% CI 0.55–3.25]). Conversely, UCM seems to increase the risk of respiratory distress syndrome (RR = 1.54 [95% CI 1.03–2.29]), compared to the control groups. In infants born at <33 weeks, UCM was associated with a reduced risk of transfusion (RR= 0.81 [95%CI 0.66–0.99]), as well as higher quantities of hemoglobin (PWMD= 0.91 g/L[95%CI 0.50–1.32]). UCM reduces the risk of transfusion in preterm infants, and increases initial hemoglobin, hematocrit, and mean blood pressure levels with respect to controls.

## 1. Introduction

Placental transfusion is the transfer of residual placental blood to the baby during birth and umbilical cord clamping. This transfer is part of the physiological transition from fetal to neonatal circulation [1].

The placenta can contain up to 40% of fetal blood volume [2]. In full-term infants, when delayed umbilical cord clamping (DCC) is performed, an additional 80–100 mL of blood is transferred and can contribute one third to one quarter of neonatal blood volume at birth [3]. In preterm infants, a randomized study of DCC versus immediate umbilical cord clamping (ICC) found an 18% increase in blood volume in the DCC group [4].

This is why the benefits and risks derived from the different ways of managing the umbilical cord in infants have been studied. With DCC, the observed effects include an increase in hemoglobin levels, reduced need for transfusion, an increase in iron deposits, and reduced rates of necrotizing enterocolitis [5,6,7].

Clinical practice guidelines (CPG) [8,9] and various scientific associations [10,11,12] recommend DCC in all births, whenever possible, due to its positive impact on neonatal health. Occasionally, it is not possible to perform DCC for varying reasons, such as immediate neonatal resuscitation or maternal hemodynamic instability. Umbilical cord milking (UCM) has been suggested as an alternative to DCC in these cases. This technique consists of milking the umbilical cord two to four times along a 10 cm or 20 cm length of cord, from the placenta toward the newborn [13,14].

In 2015, a meta-analysis was published on the use of UCM which included seven randomized controlled trials (RCTs) [13]. The control groups were made up of infants who had received DCC or ICC. One study included in this meta-analysis was on full-term infants. The authors concluded that UCM in preterm infants resulted in higher levels of hemoglobin and hematocrit than in other types of clamping, and also found a reduced risk of oxygen being needed and a reduced risk of intraventricular hemorrhage if UCM was performed. Furthermore, in 2018, another meta-analysis was published including only two RCTs of preterm infants comparing the practice of UCM with DCC. The authors concluded that UCM may reduce intraventricular hemorrhage compared to DCC [15].

Despite the demonstrated benefits of UCM for preterm infants, it is still not standard practice in delivery care and requires a new review due to the large number of RCTs published in recent years [16,17,18,19,20,21,22,23,24,25,26]. It would be especially interesting to determine the benefits and risks by gestational age and by type of clamping (DCC or ICC) in the most significant variables.

Therefore, the main objective of this systematic review and meta-analysis was to evaluate the effects of UCM in infants born at less than 37 weeks’ gestation. The secondary objective was to evaluate the effects of UCM stratified by gestational age (<33/≥33 weeks) and type of clamping (ICC/DCC).

## 2. Materials and Methods

This systematic review with meta-analysis was done in accordance with the preferred reporting items for systematic review and meta-analyses (PRISMA) declaration [27].

### 2.1. Data Sources and Searches

The search strategy was: (stripping OR milking OR squeezing) AND (umbilicus OR umbilical cord OR cord). A systematic search was performed of main database: Cochrane Library Plus, EMBASE, Scopus, PubMed, and ClinicalTrials.gov. The specific search strategy adapted to each database is provided in detail in Appendix A (Table A1).

The inclusion criteria were: (I) the type of study: RCT; (II) the population; including infants born at <37 weeks gestational age (GA).We made an initial decision to study three populations (<37, <33, and ≥33 weeks GA) separately because the effects and the results of interest would be different for these two groups; and (III) the type of procedure, where we included studies that compared UCM with a control procedure (ICC or DCC). The exclusion criteria were RCTs that included both preterm and full-term infants without the possibility of obtaining separate information for each group.

RCTs were selected with no time or language restrictions. Two reviewers (IOE and JRA) independently evaluated the articles obtained from a literature search done using titles and summaries, in an initial stage. They then evaluated the full texts that had been selected. Any dispute was resolved by reaching a consensus. If this was not possible, a third reviewer (AHM) evaluated the articles.

The main outcome of our study was neonatal mortality before discharge from hospital and the secondary results were adaptation at birth variables (cord arterial pH, Apgar score at 1 and 5 min) and hematological variables (first hematocrit and hemoglobin levels measured within the first 24 h after birth, the need for red blood cell transfusion before being discharged, peak serum bilirubin, and hyperbilirubinemia requiring phototherapy). We also included mean blood pressure within the first 6h after birth and short-term morbidities such as respiratory distress syndrome, hypotension in the first 24 h after birth requiring volume or inotropic support, intraventricular hemorrhage (any grade), need for oxygen at 28 days, necrotizing enterocolitis, sepsis, retinopathy of prematurity, patent ductus arteriosus, and duration of hospital stay.

### 2.2. Data Extraction and Quality Assessment

The three reviewers (IOE, AAA, and AHM) compiled the data and evaluated the quality independently. For the continuous outcomes, the averages and standard deviations (SD) were compiled whenever possible. When the averages and the SD were not available and originally appeared as the median and range or interquartile range, we attempted to contact the authors and ask for the results. When this was not possible, the results were converted to the mean and SD, using the methodology recommended in the Cochrane Handbook [28]. For the categorical outcomes, the counts of the study events were compiled.

The risk of bias in each study included was assessed using the criteria described in the Cochrane Handbook for Systematic Reviews of Interventions [29]. Seven domains were evaluated related with the risk of bias in each included study because there is evidence that these problems are associated with biased estimates of the treatment effect: (1) random sequence generation, (2) allocation concealment, (3) blinding of participants and personnel, (4) blinding of outcome assessment, (5) incomplete outcome data, (6) selective reporting, and (7) other bias. The opinions of the review authors were classified as “low risk”, “high risk”, or “uncertain risk” of bias.

### 2.3. Data Synthesis

For the categorical outcomes, relative risk (RR) was used together with confidence intervals of 95% (95% CI). Mantel–Haenszel fixed-effects models and Der Simonian–Laird random-effects models were used depending on the absence or presence of heterogeneity, respectively. The heterogeneity of the studies was estimated using I^2^ tests and Cochran’s Q. I^2^ values of <25%, 25%–50%, and >50% normally correspond to small, moderate, and large amounts of heterogeneity, respectively [30,31].

**Figure A1:**
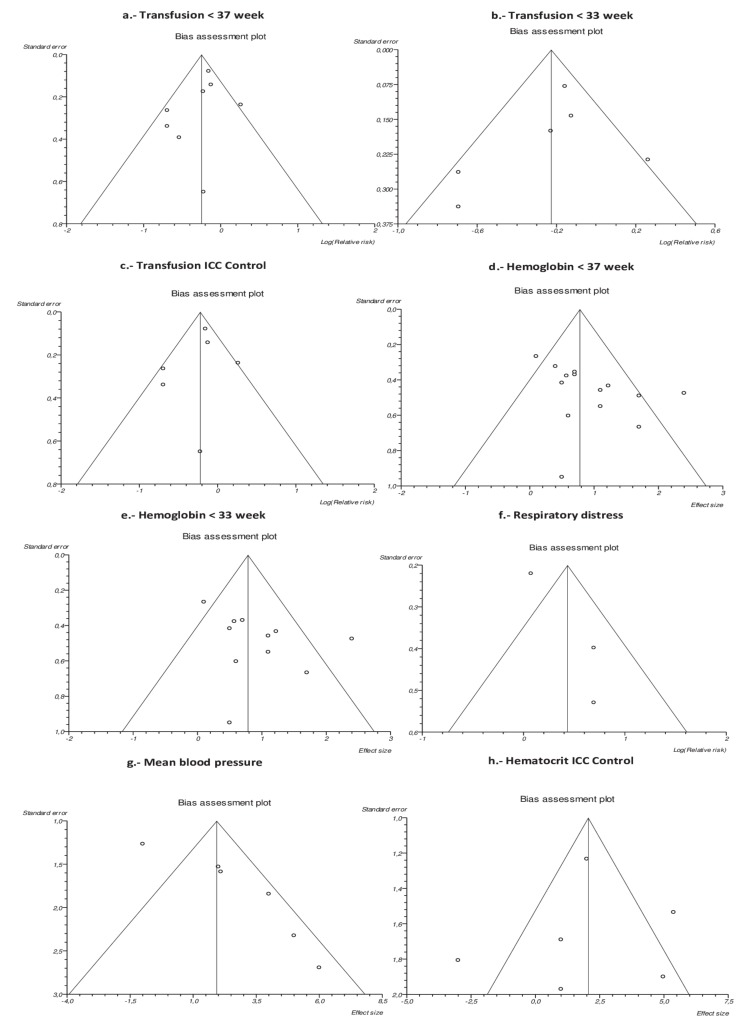
Funnel Plot.

For the quantitative outcomes, the pooled weighted mean difference (PWMD) was used with a 95% confidence interval (CI). The publication bias was also evaluated using Egger’s asymmetry test and Funnel plots (Figure A1) [32]. The statistical significance level was defined as 0.05.

All calculations were done using the statistics software StatsDirect, version 2.7.9 (StatsDirect Ltd., Cheshire, England).

### 2.4. Role of the Funding Source

The funders of the study had no role in study design, data collection, data analysis, data interpretation, writing of the report, or the decision to submit the paper for publication. All authors had full access to all data in the study and had final responsibility for the decision to submit for publication.

## 3. Results

### 3.1. Study Selection

**Figure 1:**
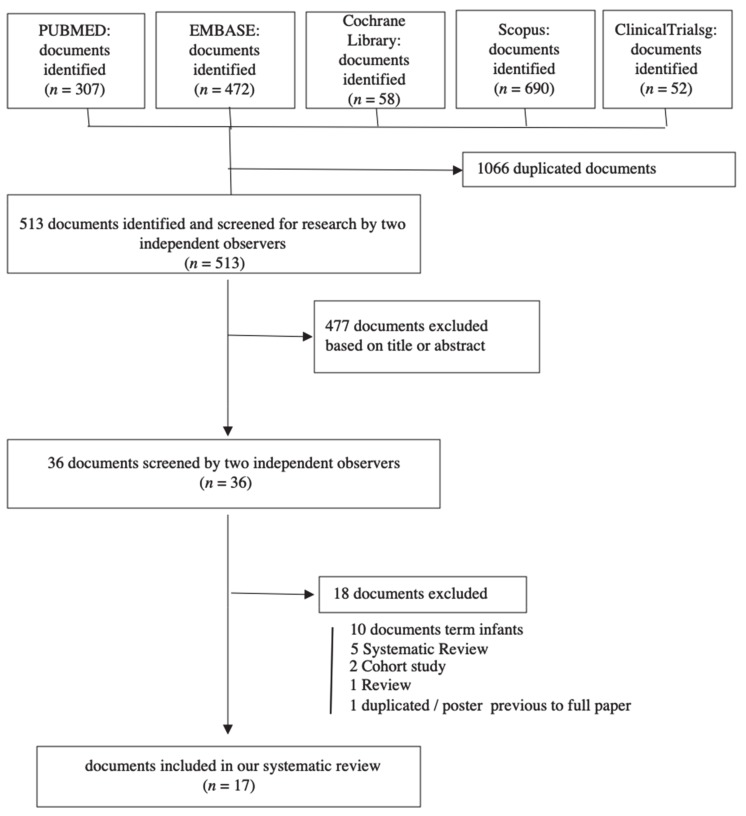
Preferred reporting items for systematic review and meta-analyses (PRISMA) flow diagram of the literature reviewing process.

A total of 1579 studies were identified in the literature search. After eliminating duplicate articles, the 477 remaining documents were screened by title and summary. After applying the inclusion/exclusion criteria, 17 articles were selected for qualitative and quantitative analysis (meta-analysis; Figure 1)

### 3.2. Study Characteristics

The sample included 2083 preterm infants with GA between 23 and <37 weeks. The selected studies were from Japan [32], the United Kingdom [33], the United States [17,22,25,26,34,35,36], Turkey [19,37], India [18,20], South Korea [21], Canada [24,26], Ireland [26], and Germany [26].

The sample size of the studies ranged from 26 to 215 infants. UCM was compared with DCC in five RCTs [17,22,25,26,36], with ICC in twelve RCTs [16,18,19,20,21,23,24,32,33,34,35,37].

The description of the UCM technique varied by study, including the number of times the cord was milked toward the baby (between two and four times) and the milking speed (between 5cm within 1s and 20 cm within 2s). 

The number of infants in each study, the description of the UCM method, how the cord was managed in the control group, and the exclusion criteria are shown in Table 1.

### 3.3. Study and Data Quality

**Figure A2:**
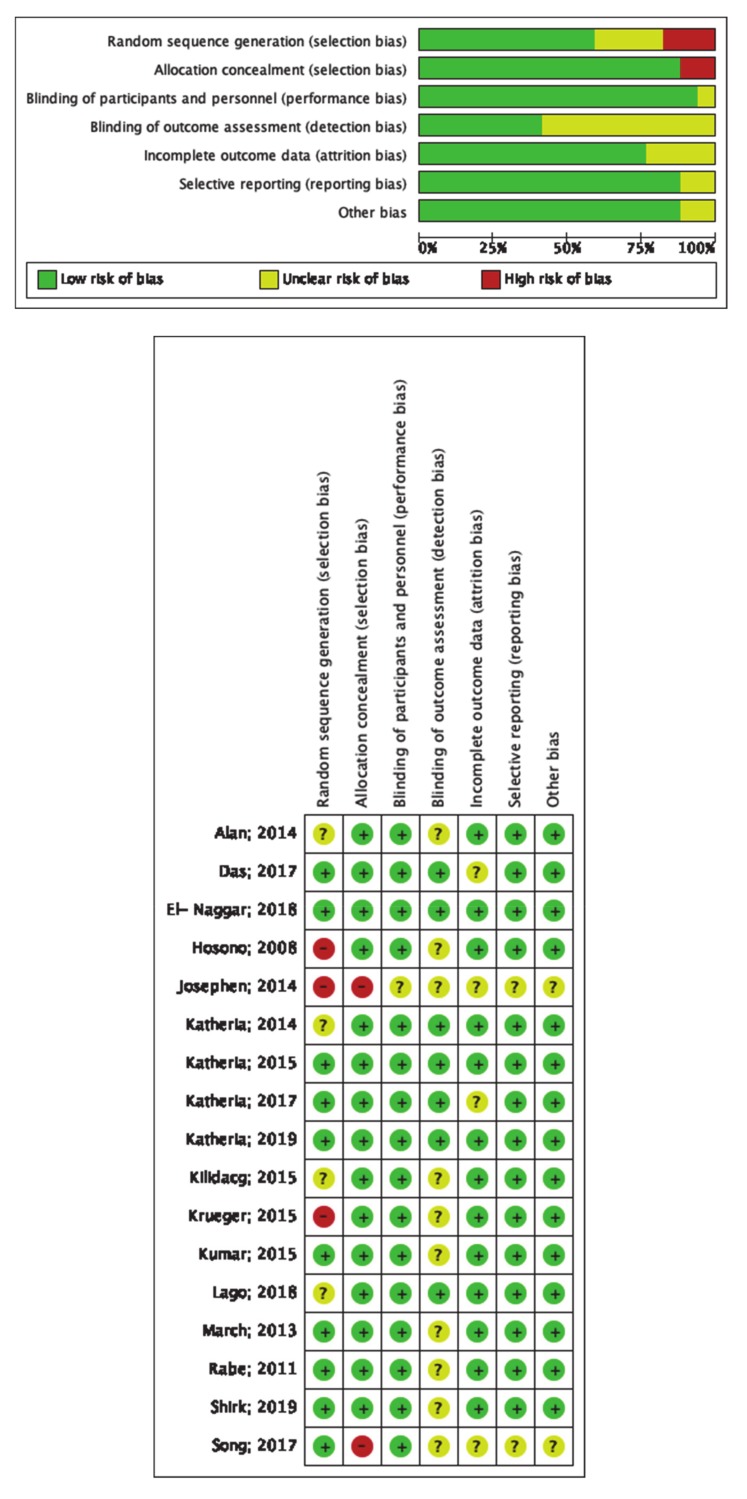
Assessment of the Risk of Bias.

The risk of bias for the seven domains of each study is shown in Figure A2. Ten of the 17studies were assessed as being low risk for random sequence generation; in four studies, the risk was not clear, and three were assessed as being high risk because the details of the methods used for randomization were not described. All of the studies stated that the health professionals involved could not be blinded due to the nature of the intervention. Table A2.

### 3.4. Meta-Analysis

#### 3.4.1. Mortality

Eleven studies [17,21,23,24,25,26,32,33,34,35,36] evaluated the risk of mortality in 1409 infants at <37 weeks GA and found no significant reduction in the risk of mortality regarding the UCM group versus the control group (RR = 0.71 [95% CI 0.47–1.08]). Similarly, in nine studies [17,21,24,26,32,33,34,35,36] that evaluated the mortality of 1067 infants at <33 weeks GA, no reduction in the risk of mortality was found in the intervention group versus the control group (RR = 0.66 [95% CI 0.43–1.03]). No significant differences were found in the sub-analysis by type of control (Table 2 and Table A3).

#### 3.4.2. Phototherapy

No significant differences were observed with regard to phototherapy between the intervention and control groups for five studies at <37 weeks GA [20,23,24,25,34] and in three studies [20,24,34] at <33 weeks GA (Table 2 and Table A3).

#### 3.4.3. Transfusion

**Figure 2:**
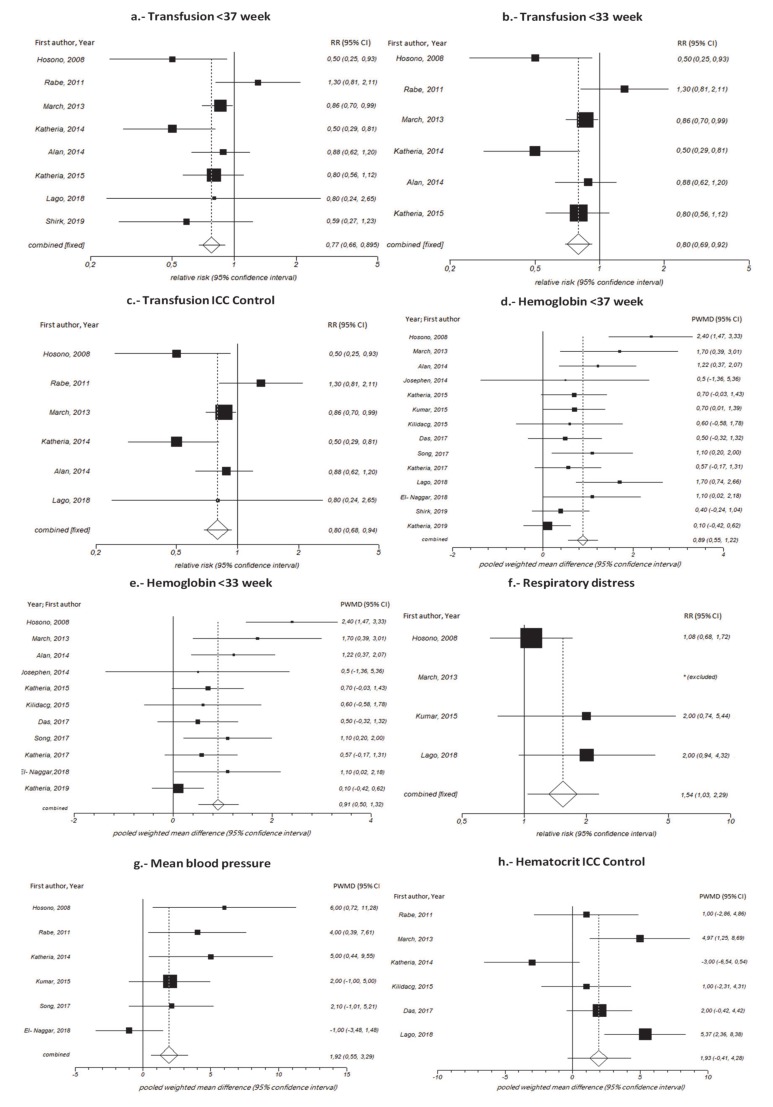
Forest plot.

Upon combining eight studies [23,25,32,33,34,35,36,37] on infants at <37 weeks GA and six studies at <33 weeks GA [32,33,34,35,36,37], we found a reduction in the risk of transfusion in the intervention groups versus the control group, with an RR of 0.78 (95% CI 0.67–0.90)and an RR of 0.81 (95% CI 0.66–0.99), respectively. The NNT (Number Needed to Treat) to avoid it was 11 (CI 95% 7–25) for those under 37 weeks and eight (CI 95% 5–19) for those under 33 weeks. Figure 2a,b.

Furthermore, when we looked at six studies that had ICC as a control group [23,32,33,34,35,37], we observed that this practice was related with a decreased risk of transfusion in the intervention groups (RR = 0.80 [95% CI 0.68–0.94];Figure 2c,Table 2, and Table A3).

#### 3.4.4. Hemoglobin

Upon grouping fourteen studies on infants born at <37 weeks GA [16,18,19,20,21,22,23,24,25,26,32,34,36,37], we observed that in the UCM group, hemoglobin levels within the first 24 h after birth were statistically higher than in the control group (PWMD =0.89 g/L [95% CI 0.55–1.22]). We also observed that in eleven studies of infants born at <33 weeks GA [16,19,20,21,22,24,26,32,34,36,37], there was an increase in hemoglobin levels in the intervention group (PWMD = 0.91 g/L [95% CI 0.50–1.32]). Likewise, we also observed that in 542 infants at >33 weeks there was an increase in hemoglobin levels in the intervention group (PWMD = 0.85 g/L [95% CI 0.17–1.53]; Figure 2d,e).

Furthermore, when we looked at 10studies that had ICC as a control group [16,18,19,20,21,23,24,32,34,37], we observed that this practice was related with increased hemoglobin compared with the intervention group (PWMD =1.14 g/L [95% CI 0.83–1.44]).

When we studied the UCM group versus the DCC group [16,22,25,26,36], we observed an increase in hemoglobin levels in the intervention group (PWMD =0.38 g/L [95% CI 0.06–0.70]; Table 2 and Table A4).

#### 3.4.5. Hematocrit

To assess the hematocrit, we examined nine studies [17,19,20,23,25,26,33,34,35] and found hematocrit levels were not higher in the UCM intervention group than in the control group (PWMD = 1.43million/mm^3^[95% CI –0.03–2.89]). However, when we studied 342infants born at >33 weeks, we observed an increase in hematocrit levels in the intervention group (PWMD=2.90 million/mm^3^[95% CI 1.28–4.52]; Figure 2h. Table 2, and Table A4).

#### 3.4.6. Peak Serum Bilirubin

No significant differences were observed in peak serum bilirubin regarding the intervention and control groups in nine studies <37 weeks GA [17,23,24,25,32,33,34,35,36] (Table 2 and Table A4).

#### 3.4.7. Mean blood pressure.

In six studies [18,21,24,32,33,35], it was observed that the mean blood pressure of the UCM group was greater than that in the control group (PWMD =2.47 mmHg [95% CI 0.39–4.55]). We did not find significant differences in the analysis by type of control and GA (Figure 2g, Table 2, and Table A4).

#### 3.4.8. Respiratory Distress Syndrome

Four studies were used to assess respiratory distress syndrome [18,23,32,34]. We observed that the risk of respiratory distress syndrome was higher in the UCM group than in the control group (RR = 1.54 [95% CI 1.03–2.29]). However, when we studied 338 infants born at >33 weeks, we observed that the risk of respiratory distress syndrome was higher in the intervention group. No significant differences were found in the sub-analysis of the type of control (Figure 2f, Table 2, and Table A3).

### 3.5. Other Variables

No inter-group differences were found for length of hospital stay, cord arterial pH, Apgar scores 1 min, Apgar scores 5 min, oxygen at birth, oxygen at 28 days, retinopathy of prematurity, using hypotensive expanders, using hypotensive drugs, necrotizing enterocolitis, patent ductus arteriosus, sepsis, and intraventricular hemorrhage (Table 2, Table A3 and Table A4).

### 3.6. Publication Bias

Publication bias was observed for the hematocrit study (Egger’s test for asymmetry; *p* =0.037), mean blood pressure (Egger’s test for asymmetry; *p* = 0.007), and for length of hospital stay (Egger’s test for asymmetry; *p* = 0.039; Table 2 and Figure A1).

## 4. Discussion

### 4.1. Main Findings

The results of our meta-analysis suggest that UCM in preterm infants may reduce the risk of transfusion and increase hemoglobin and mean arterial pressure values. The only adverse effect of UCM appears to be that it increases the risk of respiratory distress syndrome compared to control groups.

With regard to meta-analysis by gestational age, in infants born with <33 weeks of GA, UCM was associated with a reduced risk of transfusion and with higher hemoglobin levels compared to the control group. In infants born with >33 weeks, higher hematocrit levels were observed in the intervention group versus the control group.

Moreover, upon conducting the meta-analysis according to the type of controls, the only statistical differences observed was the increase in hemoglobin levels in the UCM group versus ICC and DCC.

### 4.2. Interpretation

In our review we found an increase in hemoglobin levels, which reduces the risk of anemia, as well as the need for transfusion and the complications associated with this practice [38,39]. Specifically, the improvement in hematology is due to the placenta containing approximately 15–20 mL of blood per kilogram of body weight, regardless of birth weight [2], and when performing the UCM there is an increase in systemic blood volume and, therefore, in fetal hemoglobin. These improvements in hematological values were also observed in previous studies by Al-Wassia et al. and Dang et al. [13,40].

In our study, no significant differences were found regarding intraventricular hemorrhage between the intervention group and the controls, nor when performing the sub-analysis by GA or type of clamping. However, in 2019, one study [26] was discontinued due to concerns of increased severe IVH (Intraventricular Hemorrhage) in the overall cohort and a significant difference in the premature subgroup, <28 weeks. In our study, the cut-off point of gestational age was set at <33 weeks, and previous trials included in the meta-analysis were underpowered to find such effects. Therefore, the result of this variable must be taken with caution as new trials are required to confirm these data. Therefore, until this safety is verified with other studies, this practice should not be recommended in fetuses <28 weeks.

With regard to the adaptive capacity of the infant to life, no significant differences were observed in the Apgar scores at one or five minutes, cord arterial pH, or need for oxygen at birth between the UCM group and the control groups. An increased risk of respiratory distress was observed in infants with UCM, although only four studies were included for this meta-analysis.

When we carried out sub-analysis by GA, we found a reduced need for transfusion, and in increase in hemoglobin levels in preterm infants born at <33 weeks GA. However, these improvements were not observed in the >33 weeks GA group, which is attributable to the fact that only 2–3 RCTs were included, and also to the lack of information on several of the variables included. In the sub-analysis by type of control, where we compared UCM to ICC or DCC, we can only confirm an improvement in hemoglobin levels in the UCM group compared to the other two types of controls. In this regard, new trials are needed to confirm the benefits of UCM in infants at >33 weeks GA and, especially, to compare it with DCC.

Studies are currently being conducted on animals, that have raised major concerns about the safety of UCM in lambs at 126 ± 1 days gestation (equivalent to approximately 26 weeks gestational age in humans), with spikes in blood pressure and cerebral blood flow during each milking, which are detrimental to the newborn. [41]. In this respect, more studies covering a wider range of gestational ages would be necessary to raise these concerns and thus be able to compare the different studies.

### 4.3. Strengths and Limitations

The main strength of this systematic review is the inclusion of 11 new RCTs compared with the last review published. It also analyzes the effect of UCM in different sub-populations by gestational age and type of control, as well as assessing publication bias.

One of the limitations of our systematic review is that the inclusion and exclusion criteria were very variable, therefore there is a large amount of heterogeneity in the study populations. We also observed that the variables measured varied widely between each study. Another limitation is the lack of standardization of the practice of UCM in the different studies, although the method is described in detail in the majority of them. We observed a publication bias in the result “mean blood pressure”, which could mean that the UCM group is overestimated, so the results should be interpreted with caution. On the other hand, we have not assessed the effects of UCM on severe intra-ventricular hemorrhage, and we do not have these data on long-term effects. The low number of studies that compare UCM with DCC means that is not possible to establish conclusive results.

## 5. Conclusions

The main conclusion of our systematic review is that UCM increases initial hemoglobin and mean blood pressure levels and reduces the risk of transfusion in preterm infants. There are no complications associated with this practice regarding the studied variables, except for an increased risk of respiratory distress syndrome. The UCM does not increase neonatal mortality compared to DCC and ICC procedures. In this sense, UCM could be considered as an alternative to DCC in situations where this cannot be carried out in fetuses>28 weeks, although the preferred technique should still be DCC.

## Figures and Tables

**Table 1:** Characteristics of the included studies.

Author	Year	*N*	Country	Gestational Age	UCM No. of Times	UCM Speed	Control Condition	Exclusion Criteria
Hosono et al.	2008	40	Tokyo (Japan)	24–28 wk	2–3	20 cm within 2s	ICC	Multiple births, major congenital anomalies, or chromosomal anomalies and hydrops fetalis.
Rabe et al.	2011	58	Brighton (United Kingdom)	24 ^0/7^–32^6/7^wk	4	20 cm within 2s	ICC	Multiple births, inadequate time to obtain consent before delivery, known congenital abnormalities of the fetus, rhesus sensitization, or fetal hydrops.
March et al.	2013	75	Virginia (USA)	24–28 wk	3	–	ICC	Antenatally diagnosed major fetal congenital anomaly, known Factor Rh sensitization, hydrops fetalis, known recent maternal exposure to parvovirus, elevated peak systolic velocity of the fetal middle cerebral artery, or clinical suspicion of placental abruption at delivery due to excessive maternal bleeding or uterine hypertonicity.
Katheria et al.	2014	60	San Diego (USA)	23^0/7^–31^6/7^wk	2	20 cm within 2s	ICC	Monochorionic multiples, incarcerated mothers, placenta previa, concern for abruptions, or refusal to perform the intervention by the obstetrician.
Alan et al.	2014	44	Ankara (Turkey)	<32 wk	3	5 cm within 1s	ICC	Suspected twin to twin transfusion syndrome or discordant twins, major congenital anomalies or chromosomal anomalies, vaginal bleeding due to placenta previa or abruption or placental tear, hemolytic disease of the fetus and newborn such as rhesus sensitization, intrauterine growth restriction, maternal gestational diabetes treated with insulin, hydrops fetalis; and refused parental consent.
Josephen et al.	2014	26	–	24–26^6/7^wk	3	–	ICC	Multiple gestation, congenital abnormalities, hydrops fetalis, and known fetal anemia.
Krueger et al.	2015	67	South Alabama (USA)	22–31^6/7^wk	4	–	DCC	The fetus had known anomalies or there was a suspected placental abruption.
Kumar et al.	2015	200	Northern India	32^0/7^–36^6/7^wk	3	10 cm within 1s	ICC	Umbilical cord length less than 25 cm, or were non-vigorous at birth, multiple births (twins, triplets), those born to Rh negative or retrovirus positive mothers, hydrops fetalis and those with major congenital anomalies, cord prolapse or cord anomalies like true knots were also excluded. Babies born to mothers with complications such as placental abruption, placental implantation disorders (placenta previa or accreta), or chorioamnionitis were excluded only if they were born limp.
Kilicdag et al.	2015	54	Istanbul (Turkey)	≤32 wk	4	20 cm within 2s	ICC	Congenital anomalies, placenta abruption, intrauterine growth restriction, twin–twin transfusion syndrome, discordant twin growth, vaginal delivery, and Rh hemolytic disease.
Katheria et al.	2015	154	San Diego (USA)	23^0/7^–31^6/7^wk	4	20 cm within 2s	DCC	Monochorionic multiples, incarcerated mothers, placenta previa, concern for abruptions, Rh sensitization, hydrops, congenital anomalies, or the obstetrician declining to perform the intervention.
Daset al	2017	215	Northern India	30–33 wk	2	–	ICC	Pregnant women with multiple pregnancies, suspected or proven major congenital malformation in the fetus, and antenatally diagnosed hydrops fetalis.
Song et al.	2017	66	Chungnam (South Korea)	24^0/7^–32^6/7^wk	4	20 cm within 2s	ICC	Multiple gestations, rhesus sensitization, fetal hydrops, or major fetal anomalies, and women without antenatal written consent.
Katheria et al.	2017	135	San Diego (USA)	23^0/7^–31^6/7^wk	3	–	DCC	Monochorionic multiples, incarcerated mothers, placenta previa, concern for placental abruption, Rh sensitization, hydrops, and congenital anomalies.
Lago et al.	2018	138	–	24^0^–36^6^wk	4	–	ICC	Umbilical cord abnormalities (true and false knots, short cord, nuchal cords), major congenital anomalies or chromosomal anomalies, hydrops fetalis twin–twin transfusion syndrome, clinical suspicion or diagnosis of placental abruption, and infants whose parents refused to consent.
El-Naggar et al.	2018	73	Halifax (Canada)	24–31 wk	3	10 cm within 1s	ICC	Monochorionic twins, major congenital anomalies, placental abruption, fetal anemia, and intention to withhold resuscitation.
Shirk et al.	2019	204	Cincinnati (USA)	23^0^–34^6^wk	4	–	DCC	Congenital anomalies that had been identified on prenatal sonography (not including trisomy markers), those with precipitous delivery that prevented completion of the protocol, placental abruption at the time of/or as the indication for delivery, uterine rupture, infants known to be at risk of anemia (i.e., parvovirus B19 infection and allo/isoimmunization), or patient delivered at outside institution after random assignment.
*Katheria* *et al.*	*2019*	*474*	*9 participating sites (6 in the United States and 1 site in Ireland, Germany, and Canada*)	*<32* *wk*	*3*	*20 cm within 2s*	*DCC*	*Major congenital anomalies, severe placental abruption, transplacental incision, umbilical cord prolapse, hydrops, bleeding accreta, monochorionic multiple births, fetal or maternal risk for severe compromise at delivery, and family unlikely to return for 24month neurodevelopmental testing.*

Abbreviations: wk, weeks; ICC, immediate cord clamping; DCC, delayed cord clamping; UCM, umbilical cord milking.

**Table 2:** Comparison of umbilical cord milking vs. control intervention.

Outcome	Gestational Age	No of Studies	No of Participants	RR (95% CI)b	PWMD (95% CI)b	* I * 2 Value, %	Cochran’s Q	Egger Bias
Mortality	<37 weeks	11	1.409	0.71 (0.47–1.08)		0% (0–52.7)	0.482	0.165
	<33 weeks	9	1.067	0.66 (0.43–1.03)		0% (0–54.4)	0.455	0.166
	≥33 weeks	NC	NC	NC		NC	NC	NC
	ICC control	7	510	0.51 (0.26–1.06)		0% (0–61)	0.687	0.073
	DCCcontrol	4	899	0.87 (0.52–1.47)		23.3% (0–74.8)	0.271	0.177
Transfusion	<37 weeks	8	767	**0.78 (0.67–0.90)**		42.1% (0–72.9)	0.098	0.345
	<33 weeks	6	425	0.80 (0.69–0.92) *		**53% (0–79.3)**	0.059	0.483
	≥33 weeks	2	342	0.64 (0.33–1.23)		NC	0.680	NC
	ICC control	6	409	**0.80(0.68–0.94)**		**53% (0–79.3**)	0.059	0.567
	DCC control	2	358	–0.08 (–0.16–0.00)		NC	0.621	NC
Hemoglobin	<37 weeks	14	1.830		0.89 (0.55 to 1.22) *	**53% (0–73)**	**0.01**	**0.037**
	<33 weeks	11	1.288		0.91 (0.50 to 1.32) *	**56% (0–76)**	**0.012**	0.104
	≥33 weeks	3	542		0.85 (0.17 to 1.53) *	**59.6% (0–86.6)**	0.084	NC
	ICC control	10	863		**1.14 (0.83 to 1.44)**	36.4% (0–68.5)	0.117	0.716
	DCC control	4	967		**0.38 (0.06 to 0.70)**	0% (0–67.9)	0.549	**0.011**
Phototherapy	<37 weeks	5	687	1.03 (0.92–1.15)*		**81.9% (46.5–90.5)**	**0.001**	0.208
	<33 weeks	3	345	0.99 (0.95–1.02)		17.1% (0–77.2)	0.299	NC
	≥33 weeks	2	342	1.15 (1.00–1.31)		NC	0.033	NC
	ICC control	4	483	1.06 (0.89–1.26)*		**90.2% (76–94.5)**	**<0.001**	0.294
	DCC control	1	204	1.00 (0.89–1.12)		NC	NC	NC
Hematocrit	<37 weeks <33 weeks >33 weeks ICC control DCC control	9 7 2 6 3	804 700 342 533 745		1.43 (–0.03 to 2.89)* 0.57 (–0.41 to 1.55) **2.90 (1.28 to 4.52)** 1.93 (–0.41 to 4.28) * 0.61 (–0.48 to 1.70)	**61.2% (0–79.5)** 46.1% (0–75.6) NC **67.5% (0–84.3)** 22.3% (0–78.4)	**0.008** 0.084 0.057 **0.009** 0.276	0.526 0.616 NC 0.732 NC
Respiratory distress syndrome	<37 weeks <33 weeks >33 weeks ICC control DCC control	4 NC 2 NC NC	453NC338NCNC	**1.54 (1.03–2.29)** NC **2 (1.07–3.73)** NC NC		39.6% (0–82.3) NC NC NC NC	0.191 NC >0.999 NC NC	NC NC NC NC NC
Intraventricular hemorrhage	<37 weeks<33 weeks >33 weeks ICC control DCC control	13 11 2 8 5	1.713 1.233 342 345 518	0.93 (0.76–1.15) 0.97 (0.77–1.20) 0.72 (0.38–1.38) 0.83 (0.6–1.16) 1 (0.77–1.31)		11.1% (0–54.1) 20.3% (0–60.5) NC 15.8% (0–62.8) 0% (0–64.1)	0.334 0.25 0.58 0.305 0.413	0.787 0.814 NC 0.271 0.447
Peak serum bilirubin	<37 weeks	9	869		0.11 (–0.18 to0.40)	35% (0–69)	0.138	0.313
Mean blood pressure	<37 weeks	6	497		**1.92 (0.55 to 3.25)**	**52.9% (0–79.3)**	0.059	**0.007**
Length of hospital stay	<37 weeks	5	308		–1.92 (–8.44 to 4.60)	24.1% (0–72.1)	0.260	**0.039**
Cord arterial pH	<37 weeks	4	380		–0.03 (–0.05 to 0.01)	0% (0–67.9)	0.705	0.969
Apgar scores 1 min	<37 weeks	8	756		0.02 (–0.06 to 0.10)	0.0% (0–56.3)	0.455	0.1
Apgar scores 5 min	<37 weeks	9	766		0.02 (–0.31 to 0.35)*	**72.1% (33.9–84.2)**	**0.001**	0.177
Oxygen at birth	<37 weeks	3	293	1.01 (0.82–1.23)*		**67.2% (0–88.4)**	**0.047**	NC
Oxygen at 28 days	<37 weeks	2	212	1.20 (0.67–2.14)		NC	0.375	NC
Retinopathy of prematurity	<37 weeks	9	1.204	0.80 (0.63–1.03)		35.2% (0–69.1)	0.136	0.688
Hypotensive expanders	<37 weeks	2	133	1.00 (0.53–1.88)		NC	0.475	NC
Hypotensive drugs	<37 weeks	6	547	0.70 (0.48–1.03)		7.3% (0–63.8)	0.370	0.299
Necrotizing enterocolitis	<37 weeks	10	1.477	0.71 (0.45–1.12)		0% (0–52.7)	0.920	0.315
Patent ductus arteriosus	<37 weeks	4	725	1.04 (0.78–1.40)		11.7% (0–71.5)	0.334	0.595
Sepsis	<37 weeks	9	1.237	0.96 (0.77–1.19)		0% (0–54.4)	0.615	0.205

NC, Not calculated; CI, Confidence Interval; RR, relative Risk; PWMD, pooled weighted mean difference; PWMD, pooled weighted mean difference; *,Random effects (DerSimonian–Laird). Bold means statistically significant differences.

## Appendix A

**Table A1:** Search strategies.

Database	Search Strategies	Hits
Pubmed	(stripping[All Fields] OR milking[All Fields] OR squeezing[All Fields]) AND ((“umbilicus”[MeSH Terms] OR “umbilicus”[All Fields]) OR (“umbilical cord”[MeSH Terms] OR (“umbilical”[All Fields] AND “cord”[All Fields]) OR “umbilical cord”[All Fields]) OR (“cone-rod dystrophies”[MeSH Terms] OR (“cone-rod”[All Fields] AND “dystrophies”[All Fields]) OR “cone-rod dystrophies”[All Fields] OR “cord”[All Fields]))	307
Embase	(‘stripping’/exp OR stripping OR ‘milking’/exp OR milking OR squeezing) AND (‘umbilicus’/exp OR umbilicus OR ‘umbilical cord’/exp OR ‘umbilical cord’ OR ((‘umbilical’/exp OR umbilical) AND cord) OR cord)	472
Scopus	**(*****stripping*****OR*****milking*****OR*****squeezing*****) AND (*****umbilicus*****OR*****umbilical*** AND ***cord*** **OR** ***cord*****)**	690
Clinical trials	(stripping OR milking OR squeezing) AND (umbilical cord OR cord)	52
Cochrane Library plus	(*stripping* OR *milking* OR *squeezing*) AND (*umbilicus* OR *umbilical* AND *cord* OR *cord*)	58

**Table A2:** Assessment of the risk of bias. Information sources comments by reviewers.

	Random Sequence Generation (Selection Bias)	Allocation Concealment (Selection Bias)	Blinding of Participants and Personnel (Performance Bias)	Blinding of Outcome Assessment	Incomplete Outcomes Data (Attrition Bias)	Selective Reporting (Reporting Bias)	Other Bias
**Hosono S; 2008** Randomized controlled trial Jan 2001–Dec 2002 *N* = 40 (20:20)	**High Risk**	**Low Risk**	**Low Risk**	**Unclear**	**Low Risk**	**Low Risk**	**Low Risk**
	No information	Serially numbered opaque envelopes	Not blinded: Clinicians	The variables measured as principal outcomes do not depend on blinding	No missing values Follow up: Not stated (but at least 84 days according to the Kaplan-Meier curves plot)		
**Rabe H; 2011** Randomized controlled trial No date *N* = 58 (31:27)	**High Risk**	**Low Risk**	**Low Risk**	**Unclear**	**Low Risk**	**Low Risk**	**Low Risk**
	Randomization was based on computer-created tables performed by a person not involved in the trial (stratified by gestational age)	Sealed opaque envelopes and consecutively numbered.	Not blinded: Clinicians	The variables measured as principal outcomes do not depend on blinding	No missing values Follow up: 42 days		According to sample size calculation, they need 58 (29 in each group and then 27 and 31, possible poor randomization or loss of random sequence masking
**March MI, 2013** Randomized controlled trial Sep 2009–Jun 2011 *N* = 75 (36:39)	**High Risk**	**Low Risk**	**Low Risk**	**Unclear**	**Low Risk**	**Low Risk**	**Low Risk**
	Were randomized before delivery to one of two groups using random permuted blocks of 10; an independent statistician provided the randomization sequence	Serially numbered opaque envelopes contained arm bands	Neonatologists and pediatric support staff were not blinded to treatment assignment	They were not alerted for study participation or treatment assignment and no notation of study participation	Control 18 missing values Milking 20 missing values Follow up: 28 days		
**Katheria A; 2014** Randomized controlled trial Feb 2011–Jan 2013 *N* = 60 (30:60)	**High Risk**	**Low Risk**	**Low Risk**	**Unclear**	**Low Risk**	**Low Risk**	**Low Risk**
	Infants were randomized by the placement of their information in opaque, sealed envelopes. The randomization cards assigned a subject identification number that was kept by the research coordinator	In opaque, sealed envelopes	Not blinded: Clinicians	Blinded serial echocardiographic examinations were performed	No missing values		
**Alan S; 2014** Randomized controlled trial Apr 2011–Feb 2013 *N* = 44 (22:22)	**High Risk**	**Low Risk**	**Low Risk**	**Unclear**	**Low Risk**	**Low Risk**	**Low Risk**
	Subjects were randomly assigned to one of the two experimental groups	Sequentially numbered sealed non-transparent envelopes	The intervention was unmasked for the attending neonatal and obstetric teams in the delivery room	Nothing stated. Some principal variables do not depend on blinding	Five missing values in each group Follow up: 28 days		
**Josephen; 2014** Randomized controlled trial Up to Aug 2013 *N* = 26 (13:13)	**High Risk**	**Low Risk**	**Low Risk**	**Unclear**	**Low Risk**	**Low Risk**	**Low Risk**
	No information	No information	No information	Nothing stated. Some principal variables do not depend on blinding	No information	No information	No information
**Katheria A; 2015** Randomized controlled trial *N* = 154 (75:79)	**High Risk**	**Low Risk**	**Low Risk**	**Unclear**	**Low Risk**	**Low Risk**	**Low Risk**
	Computer-generated randomization	Opaque, sealed envelopes	The blinding was achieved by allowing only the nurse attending the delivery and the obstetrician performing the intervention to be aware of the allocation arm	Blinded echocardiograms and head ultrasounds were performed	No missing values Follow up: 24 h		
**Krueger MS; 2015** Prospective randomized trial Aug 2012–Nov 2013 *N* = 67 (32:35)	**High Risk**	**Low Risk**	**Low Risk**	**Unclear**	**Low Risk**	**Low Risk**	**Low Risk**
	An equal number of envelopes were created for each arm and were scrambled by a third-party registered nurse	Opaque envelopes	The neonatal team was not told which patients were participating in the study, and the randomization arm was not documented on the infants’ charts	Nothing stated. Some principal variables do not depend on blinding. Some secondary outcomes may be subjective. May have blinded the assessor	No missing values		
**Kumar B; 2015** Randomized controlled trial 2013–14 *N* = 200 (100:100)	**High Risk**	**Low Risk**	**Low Risk**	**Unclear**	**Low Risk**	**Low Risk**	**Low Risk**
	Used an online generated random number list	Sealed envelope was opened by a delivery room staff nurse	Open-label	Nothing stated. Some variables may be subjective. They may have blinded the analyst	Control: 14 missing values Experimental: nine missing values Follow up: six weeks		
**Kilicdag H, 2015** Prospective randomized study Aug 2012–Aug 2013 *N* = 54 (25:29)	**High Risk**	**Low Risk**	**Low Risk**	**Unclear**	**Low Risk**	**Low Risk**	**Low Risk**
	Were randomly assigned to one of the two groups	Using sequentially numbered sealed non-transparent envelopes	No information	Nothing stated. Some variables may be subjective, but it is not clear (segments, bands).If not subjective then low risk. Still, they may have blinded the analyst	Follow up: seven days		
**Das S; 2017** Randomized controlled trial Nov 2012–Dec 2013 *N* = 215 (116:99)	**High Risk**	**Low Risk**	**Low Risk**	**Unclear**	**Low Risk**	**Low Risk**	**Low Risk**
	Random sequence was generated using a secure web-based randomization algorithm	Serially numbered, sealed, and opaque envelopes	Blinding of the personnel and participants was not feasible due to the nature of intervention	The laboratory person who analyzed the serum ferritin levels was blinded to group allocation	Follow up: three months		
**Song S; 2017** Randomized controlled trial Mar 2012–Jun 2015 *N* = 66 (34:32)	**High Risk**	**Low Risk**	**Low Risk**	**Unclear**	**Low Risk**	**Low Risk**	**Low Risk**
	Randomized by computer-generated random numbers just before delivery	No information	No information	No information	No information	No information	No information
**Katheria A; 2017** Randomized controlled trial Aug 2013–Aug 2014 *N* = 197 (99:98)	**High Risk**	**Low Risk**	**Low Risk**	**Unclear**	**Low Risk**	**Low Risk**	**Low Risk**
	Using computer-generated allocation	Sealed envelopes before delivery	The blinding was achieved by allowing only the ALS nurse attending the delivery and the obstetrician performing the intervention to be aware of the allocation arm	Neurodevelopmental assessment was carried out by examiners who were trained in administration of the Bayley-III, had excellent inter-rater reliability (0.90), and masked to the umbilical cord milking or DCC status	Milking: 23 missing values (a lot)DCC: 25 missing values (a lot) Follow up: two years		
**Lago;2018** Randomized controlled trial Feb 2013–Apr 2016 *N* = 138 (69:69)	**High Risk**	**Low Risk**	**Low Risk**	**Unclear**	**Low Risk**	**Low Risk**	**Low Risk**
	Patients were randomized into one of the two groups: MM or ECC.	Randomization was carried out by a sealed non-transparent envelope and took place just prior to birth	The neonatologist was not aware of the timing of cord clamping. Special attention was paid to mask the intervention to the neonatology staff members (both physician and nurses) who were at the delivery room or theatre giving the first clinical care to the infants	Addition, the cord clamping interval was not registered in the infant’s chart, so this information was not available for the staff in the neonatal intensive care unit (NICU).	21 missing values or exclusions Follow up: six months		
**El-Naggar; 2018** Randomized controlled trial *N* = 73 (37:36)	**High Risk**	**Low Risk**	**Low Risk**	**Unclear**	**Low Risk**	**Low Risk**	**Low Risk**
	Was established using variable block sizes randomization table	Concealed opaque envelopes were prepared ahead of time and were opened just before the time of delivery	Despite our efforts to keep the healthcare providers blinded to the study intervention by not documenting the intervention in the charts, we cannot be absolutely sure that full blinding was achieved	Both the echocardiographer and the offline reader were blinded to patient’s assignment	No missing values Follow up: 48 h		
**Shirk; 2019** Randomized controlled trial Apr 2014–Jun 2018 *N* = 204 (104:100)	**High Risk**	**Low Risk**	**Low Risk**	**Unclear**	**Low Risk**	**Low Risk**	**Low Risk**
	The patients were assigned randomly via block randomization with an allocation ratio of 1:1	Sealed opaque envelopes	Unblinded	Nothing stated. Some variables do not depend on blinding. They may have blinded the analyst			
**Katheria; 2019** Randomized controlled trial Jun 2017–Dec 2018 *N* = 474 (236:238)	**High Risk**	**Low Risk**	**Low Risk**	**Unclear**	**Low Risk**	**Low Risk**	**Low Risk**
	Opened a sequentially numbered opaque randomization envelope from the appropriate gestational age strata (23 weeks 0 days through 27 weeks six days or 28 weeks zero days through 31 weeks six days). Randomization was computer generated using permuted block sizes of two and four and was stratified by site	Opaque envelopes prior to delivery	All outcome assessments were performed by blinded team members	Were adjudicated by two independent pediatric radiologists or neuroradiologists who were not affiliated with any of the study sites and were blinded to randomization assignment	No missing data were identified for the primary outcome of incidence of death or severe intraventricular hemorrhage at 6 months’ corrected gestational age		

Abbreviations: MM, milking maneuver; ECC, early cord clamping.

Table A3 Categorical variables under study in preterm infants.

**Table:** (**a**)

	Mortality		Hypot. Expanders		Hypot. Drugs		Respiratory distress syndrome		Necrotizing enterocolitis		Sepsis		Intraventricular hemorrhage	
Author	Milking	Control	Milking	Control	Milking	Control	Milking	Control	Milking	Control	Milking	Control	Milking	Control
2008; Hosono	2/20	3/20	NR	NR	NR	NR	14/20	13/20	NR	NR	2/20	1/20	3/20	5/20
2011; Rabe	2/27	4/31	NR	NR	NR	NR	NR	NR	1/27	4/31	1/27	0/31	3/27	7/31
2013; March	2/36	4/39	NR	NR	0/36	1/39	36/36	39/39	6/36	10/39	10/36	18/39	9/36	20/39
2014; Katheria	2/30	1/30	11/30	12/30	10/30	10/30	NR	NR	NR	NR	NR	NR	8/30	11/30
2014; Alan	NR	NR	NR	NR	NR	NR	NR	NR	2/19	1/19	16/19	16/19	3/19	0/19
2014; Josephen	NR	NR	NR	NR	NR	NR	NR	NR	NR	NR	NR	NR	NR	NR
2015; Katheria	2/75	6/79	NR	NR	NR	NR	NR	NR	1/75	0/79	5/75	3/79	5/75	10/79
2015; Krueger	0/35	3/32	NR	NR	NR	NR	NR	NR	NR	NR	NR	NR	5/35	4/32
2015; Kumar	NR	NR	NR	NR	NR	NR	10/100	5/100	NR	NR	NR	NR	NR	NR
2015; Kilicdag	NR	NR	NR	NR	NR	NR	NR	NR	NR	NR	NR	NR	NR	NR
2017; Das	NR	NR	NR	NR	NR	NR	NR	NR	1/107	1/90	7/107	8/90	6/107	3/90
2017; Song	2/34	9/32	NR	NR	10/34	20/32	NR	NR	0/34	1/32	23/34	25/32	NR	NR
2017; Katheria	NR	NR	NR	NR	8/70	12/65	NR	NR	NR	NR	4/70	1/65	9/70	6/65
2018; Lago	0/69	0/69	NR	NR	3/69	1/69	16/69	8/69	2/69	1/69	NR	NR	4/69	4/69
2018; El-Naggar	1/37	1/36	1/37	0/36	1/37	0/36	NR	NR	4/37	4/36	NR	NR	13/37	10/36
2019; Shirk	5/100	4/104	NR	NR	NR	NR	NR	NR	3/100	6/104	NR	NR	10/100	16/104
2019;Katheria	17/236	15/238	NR	NR	NR	NR	NR	NR	8/236	13/238	25/236	24/238	57/236	50/238
Egger Bias (*p*-value)	0.165		NC		0.299		NC		0.315		0.205		0.787	
I^2^ 95% CI	0% (0–52.7)		NC		7.3% (0–63.8)		39.6% (0–82.3)		0% (0–52.7)		0% (0–54.4)		11.1% (0–54.1)	
Cochran’s Q (*p*-value)	0.482		0.475		0.370		0.191		0.920		0.615		0.334	
RR 95% CI	0.71 (0.47–1.08)		1 (0.53–1.88)		0.70 (0.48–1.03)		**1.54 (1.03**–**2.****29)**		0.71 (0.45–1.12)		0.96 (0.77–1.19)		0.93 (0.76–1.15)	

RR: Relative Risk. NR: Non reported; NC Not calculated; CI: Confidence Interval. Bold means statistically significant differences.

**Table:** (**b**)

	Transfusion		Phototherapy		Oxygen at Birth		Oxygen at 28 Days		Patent Ductus Arteriosus		Retinopathy of Prematurity	
Author	Milking	Control	Milking	Control	Milking	Control	Milking	Control	Milking	Control	Milking	Control
2008; Hosono	7/20	14/20	NR	NR	NR	NR	NR	NR	5/20	7/20	8/20	17/20
2011; Rabe	17/27	15/31	NR	NR	NR	NR	4/27	6/31	NR	NR	1/27	0/31
2013; March	30/36	38/39	33/36	38/39	NR	NR	NR	NR	NR	NR	28/36	31/39
2014; Katheria	11/30	22/30	NR	NR	NR	NR	NR	NR	NR	NR	NR	NR
2014; Alan	15/19	17/19	NR	NR	NR	NR	NR	NR	NR	NR	NR	NR
2014; Josephen	NR	NR	NR	NR	NR	NR	NR	NR	NR	NR	NR	NR
2015; Katheria	31/75	41/79	NR	NR	57/75	56/79	16/75	12/79	NR	NR	1/75	2/79
2015; Krueger	NR	NR	NR	NR	NR	NR	NR	NR	NR	NR	6/35	4/32
2015; Kumar	NR	NR	NR	NR	NR	NR	NR	NR	NR	NR	NR	NR
2015; Kilicdag	NR	NR	NR	NR	NR	NR	NR	NR	NR	NR	NR	NR
2017; Das	NR	NR	104/107	89/90	NR	NR	NR	NR	NR	NR	8/107	3/90
2017; Song	NR	NR	NR	NR	28/34	31/32	NR	NR	NR	NR	0/34	2/32
2017; Katheria	NR	NR	NR	NR	NR	NR	NR	NR	NR	NR	NR	NR
2018; Lago	4/69	5/69	41/69	25/69	NR	NR	NR	NR	14/69	7/69	NR	NR
2018; El-Naggar	NR	NR	36/37	33/36	31/37	26/36	NR	NR	12/37	10/36	2/37	3/36
2019; Shirk	9/100	16/104	85/100	88/104	NR	NR	NR	NR	NR	NR	NR	NR
2019; Katheria	NR	NR	NR	NR	NR	NR	NR	NR	42/236	46/238	10/236	19/238
**Egger Bias (*p*-value)**	0.345		0.208		NC		NC		0.595		0.688	
**I^2^ 95% CI**	42.1% (0–72.9)		**81.9% (46.5–90.5**)		**67.2% (0–88.4)**		NC		11.7% (0–71.5)		35.2% (0–69.1)	
**Cochran’s Q (*p*-value)**	0.098		**<0.001**		**0.047**		0.375		0.334		0.136	
**RR 95% CI**	**0.78 (0.67–0.90)**		1.03 (0.92–1.15)*		1.01 (0.82–1.23)*		1.20 (0.67–2.14)		1.04 (0.78–1.40)		0.80 (0.63–1.03)	

RR: Relative Risk. NR: Non reported; NC: Not calculated; CI: Confidence Interval. *Random effects (DerSimonian–Laird). Bold means statistically significant differences.

**Table A4:** Quantitative variables under study in preterm infants.

	Hemoglobin		Hematocrit		Mean Blood Pressure		Length of Hospital Stay		Cord Arterial pH		Peak Serum Bilirubin, Median		Apgar Scores 1 min		Apgar Scores 5 min	
Author	Milking	Control	Milking	Control	Milking	Control	Milking	Control	Milking	Control	Milking	Control	Milking	Control	Milking	Control
2008; Hosono	16.5 (1.4)	14.1 (1.6)	NR	NR	34.0 (9.0)	28.0 (8.0)	NR	NR	NR	NR	8.18 (2.57)	7.83 (2.45)	5.40 (2.0)	4.20 (1.80)	7.3 (1.7)	6.9 (1.7)
2011; Rabe	NR	NR	52.0 (8.0)	51.0 (7.0)	35.0 (8.0)	31.0 (6.0)	105 (75.62)	72.5 (45.76)	7.2 (0.15)	7.2 (0.09)	10.17 (3.09)	10.34 (1.81)	NR	NR	7.0 (2.00)	8.5 (0.97)
2013; March	15.43 (3.70)	13.73 (1.84)	45.6 (10.57)	40.63 (5.31)	NR	NR	NR	NR	7.3 (0.0)	7.33 (0.07)	5.16 (0.84)	5.6 (1.46)	3.5 (3.47)	3.66 (2.30)	6.16 (1.54)	6.33 (1.53)
2014; Katheria	NR	NR	43.0 (7.0)	46.0 (7.0)	41.0 (9.0)	36.0 (9.0)	NR	NR	NR	NR	8.0 (2.0)	7.0 (2.0)	NR	NR	NR	NR
2014; Alan	16.62 (1.23)	15.4 (1.62)	NR	NR	NR	NR	45.75 (14.4)	51.5 (17.28)	NR	NR	NR	NR	6.25 (1.30)	6.0 (1.57)	7.75 (0.78)	7.5 (1.04)
2014; Josephen	13.9 (2.8)	13.4 (1.8)	NR	NR	NR	NR	NR	NR	NR	NR	NR	NR	NR	NR	NR	NR
2015; Katheria	16.3 (2.4)	15.6 (2.2)	NR	NR	NR	NR	NR	NR	NR	NR	8.1 (2.9)	7.3 (2.2)	NR	NR	NR	NR
2015; Krueger	NR	NR	47.71 (4.7)	47.75 (8.3)	NR	NR	67.8 (29.0)	71.2 (33.0)	NR	NR	8.27 (2.6)	8.38 (2.6)	NR	NR	NR	NR
2015; Kumar	16.7 (2.3)	16 (2.7)	NR	NR	50 (11.4)	48 (10.2)	NR	NR	NR	NR	NR	NR	6.90 (0.30)	6.90 (0.30)	NR	NR
2015; Kilicdag	18.2 (2.3)	17.6 (2.1)	52.3 (6.1)	51.3 (6.3)	NR	NR	NR	NR	NR	NR	NR	NR	5.75 (1.23)	5.25 (1.27)	8.0 (0.49)	7.5 (1.01)
2017; Das	15.0 (2.0)	14.5 (3.0)	47.0 (7.0)	45.0 (8.0)	NR	NR	NR	NR	NR	NR	NR	NR	NR	NR	9.0 (0.00)	8.66 (0.75)
2017; Song	15.8 (1.6)	14.7 (2.1)	NR	NR	31.7 (6.2)	29.6 (6.7)	54.7 (19.3)	51.5 (44.8)	7.3 (0.9)	7.3 (0.2)	NR	NR	5.5 (2.7)	5.1 (2.4)	7.8 (1.8)	7.5 (1.7)
2017; Katheria	16.35 (2.39)	15.78 (1.94)	NR	NR	NR	NR	NR	NR	NR	NR	NR	NR	NR	NR	NR	NR
2018; Lago	17.93 (2.96)	16.23 (2.80)	54.20 (9.27)	48.83 (8.76)	NR	NR	NR	NR	NR	NR	11.09 (3.21)	11.24 (3.56)	NR	NR	NR	NR
2018; El-Naggar	16.1 (2.3)	15.0 (2.4)	NR	NR	33.0 (5.6)	34.0 (5.2)	75.66 (37.79)	77.66 (38.6)	NR	NR	9.76 (2.47)	9.01 (1.89)	4.66 (0.77)	4.66 (3.86)	7.00 (1.54)	7.00 (1.54)
2019; Shirk	17.2 (2.1)	16.8 (2.5)	51.8 (6.2)	49.9 (7.7)	NR	NR	NR	NR	7.14 (0.77)	7.23 (0.09)	8.8 (2.2)	8.8 (2.5)	6.66 (2.25)	6.66 (2.25)	8.00 (1.50)	8.33 (1.50)
2019; Katheria	16.5 (3.1)	16.4 (2.7)	48.6 (8.2)	48.6 (7.9)	NR	NR	NR	NR	NR	NR	NR	NR	NR	NR	NR	NR
**Egger Bias (*p* value)**	**0.037**		0.526		**0.007**		**0.039**		0.969		0.313		0.1		0.177	
I^2^ 95% CI	53% (0–73)		61.2% (0–79.5)		52.9% (0–79.3)		24.1% (0–72.1)		0% (0–67.9)		35% (0–69)		0 (0–56.3)		72.1 (33.9–84.2)	
**Cochran’s Q (*p*-value)**	**0.01**		**0.008**		0.059		0.260		0.705		0.138		0.455		**0.001**	
**PWMD CI 95%**	**0.89 (0.55 to 1.22) ^b^**		1.43 (–0.03 to 2.89) ^a^		**1.92 (0.55 to 3.25)**		–1.92 (–8.44 to 4.60) ^a^		–0.03 (–0.05 to–0.01) ^a^		**0.11 (–0.18 to 0.40) ^a^**		**0.02 (–0.06 to 0.1) ^a^**		0.02 (–0.31 to 0.35) ^b^	

PWMD: Pooled weighted mean difference; NR: Non reported; ^a^ Mantel–Haenszel fixed effect. ^b^ Random effects Model (DerSimonian–Laird). Bold means statistically significant differences.
